# Use of the bootstrap in analysing cost data from cluster randomised trials: some simulation results

**DOI:** 10.1186/1472-6963-4-33

**Published:** 2004-11-18

**Authors:** Terry N Flynn, Tim J Peters

**Affiliations:** 1 MRC Health Services Research Collaboration, Department of Social Medicine, University of Bristol, Canynge Hall, Whiteladies Road, Bristol BS8 2PR, UK; 2 Academic Unit of Primary Health Care, Department of Community Based Medicine, University of Bristol, Cotham House, Cotham Hill, Bristol BS6 6JL, UK

## Abstract

**Background:**

This work has investigated under what conditions confidence intervals around the differences in mean costs from a cluster RCT are suitable for estimation using a commonly used cluster-adjusted bootstrap in preference to methods that utilise the Huber-White robust estimator of variance. The bootstrap's main advantage is in dealing with skewed data, which often characterise patient costs. However, it is insufficiently well recognised that one method of adjusting the bootstrap to deal with clustered data is only valid in large samples. In particular, the requirement that the number of clusters randomised should be large would not be satisfied in many cluster RCTs performed to date.

**Methods:**

The performances of confidence intervals for simple differences in mean costs utilising a robust (cluster-adjusted) standard error and from two cluster-adjusted bootstrap procedures were compared in terms of confidence interval coverage in a large number of simulations. Parameters varied included the intracluster correlation coefficient, the sample size and the distributions used to generate the data.

**Results:**

The bootstrap's advantage in dealing with skewed data was found to be outweighed by its poor confidence interval coverage when the number of clusters was at the level frequently found in cluster RCTs in practice. Simulations showed that confidence intervals based on robust methods of standard error estimation achieved coverage rates between 93.5% and 94.8% for a 95% nominal level whereas those for the bootstrap ranged between 86.4% and 93.8%.

**Conclusion:**

In general, 24 clusters per treatment arm is probably the minimum number for which one would even begin to consider the bootstrap in preference to traditional robust methods, for the parameter combinations investigated here. At least this number of clusters and extremely skewed data would be necessary for the bootstrap to be considered in favour of the robust method. There is a need for further investigation of more complex bootstrap procedures if economic data from cluster RCTs are to be analysed appropriately.

## Background

The greater complexity of cluster randomised controlled trials (RCTs) compared with their individually randomised counterparts has led to much methodological work concerning their design and analysis[1]. However, the analysis of cost data from these trials has received little attention to date. The conceptual issues arising in this context have been explored [2] but, briefly, there are two problems.

The first is that many trials randomise only a small number of clusters. This can sometimes produce inadequate randomisations where, for example, all clusters with a characteristic related to outcome are allocated to one treatment arm [3]. It may also be difficult to make inferences on cluster level covariates and between-cluster variability. Although in theory this problem is just as relevant to clinical data, in practice methods of analysis based on the *t*-test are fairly robust to moderate violations of the assumptions of normality and homogeneity of variances [4]. The situation with highly skewed continuous economic data, however, may be more serious. Indeed, at either the individual or cluster level, skewed data are potentially very problematic, particularly with small numbers of clusters. Appeals to normality of data may not be reasonable for the distribution of cluster means, given variation in medical practice, social and geographical factors. In individually randomised trials, problems of skewness and small sample sizes have sometimes resulted in confidence intervals with poor coverage properties (such as negative lower limits for mean costs). In such circumstances economic data have been analysed using methods such as the nonparametric bootstrap [5], first proposed by Efron [6]. This relies on computer-intensive resampling methods rather than a formula and commensurate appeals to the central limit theorem. In essence, by treating the sample at hand as the population, repeated resampling *with replacement *from this 'population' and calculation of a parameter of interest builds up a picture, the 'empirical distribution' of this parameter, based on so-called '*B *bootstrap estimates' of the parameter of interest. This can be used to construct directly the required confidence interval by, for instance, reading off the 2.5% and 97.5% percentiles of the distribution. Bootstrapped confidence intervals may, therefore, be asymmetric and be better able to deal with skewed data.

The bootstrap approach is flexible but does assume that the data are independently and identically distributed [7,8]. When stratification, cluster sampling or probability weights are introduced into sampling this assumption is violated and the bootstrap as described above will give incorrect inferences. Work has been carried out in the 1980s and 1990s to generalise the bootstrap to survey sampling and regression analysis [7,9]. The bootstrap is included in some standard statistical packages, but it is often overlooked that confidence intervals from this may have poor coverage properties when there is a small number of clusters – a phenomenon that is common in cluster RCTs.

This paper details the results of simulation studies to evaluate how clustered cost data might be analysed. Small numbers of clusters together with skewed data were utilised to ascertain how the bootstrap performed against a method of analysis commonly used in clustered clinical data. Thus it details the generation and analysis of a *single outcome *model. This single outcome model was primarily conceptualised as a model for cost data and the term cost is therefore used as short-hand. However, some of the scenarios presented may be equally applicable to clinical data.

## Methods

The performances of confidence intervals for simple differences in mean costs utilising a robust (cluster-adjusted) standard error and from two cluster-adjusted non-parametric bootstrap procedures were compared in terms of confidence interval coverage. Specifically, the comparison was of the percentage of 20,000 simulations for which the estimated 95% confidence interval contained the true value of the treatment effect. If the observed coverage were to be 95% on average across the simulations, then 20,000 simulations would from simple binomial theory give a margin of error of approximately 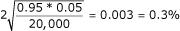, which was considered acceptable. Over 20,000 simulations per parameter set, then, the following were noted:

1. The mean value (over all simulations) of the estimated treatment effect and the confidence limits under each of the three procedures,

2. The percentages of simulations for which the estimated confidence interval did not contain the true value of the treatment effect (zero) *and *whose lower/upper limit was greater/less than this true value,

3. The percentage of simulations for which the estimated confidence interval contained the true value for the treatment effect. This figure was simply 100 minus the sum of the two percentages in 2 above.

Thus ideally the two figures in 2 should each be 2.5% whilst that for 3 should be 95% (the nominal level). It was decided to split observed non-coverage rates according to whether there was spurious positive treatment effect or a spurious negative one because skewed distributions were expected to have different implications for each of these.

### Data generation process

The data were constructed by assuming there were *n *individuals in each of *2k *clusters. Half of these were randomised to a hypothesised intervention group, whilst the other half were randomised to a control group. A random effects model incorporating a treatment dummy variable was used:

*E*_*hij *_= *α*_*hi *_+ *βT*_*h *_+ *ε*_*hij *_    *h *= 0,1; *i *= 1,...*k*;*j *= 1,...*n*;

*E*(*α*_*hi*_) = 0; *E*(*ε*_*hij*_) = 0


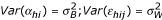


Thus the *j*th individual in the *i*th cluster was randomised to receive treatment *h*. Each individual's outcome, *E*_*hij*_, comprised three elements: the effect of treatment, *βT*_*h*_, the cluster-specific effect, *α*_*hi*_, and the individual-specific effect, *ε*_*hij*_. In order to inform the values of these parameters, it was necessary to undertake some exposition of how cost data might be distributed in a cluster RCT.

### Conceptualising costs in a cluster RCT

In attempting to construct realistic scenarios for cost data from a cluster RCT, three main factors were considered:

1. What is the nature of the distribution of individual patient costs expected to be in the population of patients normally eligible for treatment?

2. How representative of this population distribution are the cost distributions within clusters likely to be? This has implications for the ICC and the assumptions regarding the distributions.

3. How might the introduction of an intervention affect 2 above?

As detailed previously, the existence of one unrepresentative cluster (such as a London teaching hospital) in one arm may affect the ICC, independently of treatment, or the treatment could directly change the ICC and the distribution [2].

### Parameters varied in the simulation model

This section formally sets out the parameters which, when varied in the simulation model, attempted to capture two potential extreme scenarios as well as situations in between. Under the first scenario, all clusters are equally representative of the population, leading to a high degree of skewness within most, if not all, clusters. Under such a scenario, the ICC is likely to be small and results would be expected to be robust to any incorrect assumptions made regarding the between-cluster distribution. Under the second scenario, within-cluster costs are expected to be more homogenous and much of the skewness in the cost data at the population level is attributable to differences in the cluster mean costs. As a result, the ICC would be expected to be much larger, and the between-cluster distribution might be expected to exhibit considerable skewness. Six factors were varied:

1. The between-cluster distributions,

2. The within-cluster distributions,

3. The ICC in the control group,

4. The ICC in the intervention group,

5. The number of clusters in each intervention arm,

6. The number of individuals in each cluster.

### The between-cluster distributions

There were two distributional assumptions used for the cluster means. The first was the normal distribution and the second was the lognormal distribution, which, for a given mean and variance, exhibits higher kurtosis and skewness than the gamma distribution, the main alternative skewed distribution.

For each of the ICC combinations given below, there were three possible combinations of the between-cluster distributions:

1. The cluster means in both the control and intervention groups were normally distributed

2. The cluster means in both the control and intervention groups were lognormally distributed

3. The cluster means in the (nominal) control group were normally distributed, whilst in the (nominal) intervention group they were lognormally distributed.

### The within-cluster distributions

The same distribution was used for individual level data in both treatment arms in order not to further increase the number of parameter combinations possible. Lognormal data were used in order to provide a scenario applicable to individual level cost data.

### The ICC in the control group

Given that the likely range of cost ICCs is largely unknown, values between zero and 0.5 were used. While high, the upper value is consistent with the one published source of cost ICCs [10]. The ICCs used were 0.01, 0.1 and 0.25 for one of the two treatment groups. The value 0.5 was not used for the control group but was achieved in the intervention group as a result of changes in the ICC (see below). The total variance, equal to the between-cluster variance plus the within-cluster variance, was arbitrarily fixed at 100.

### The ICC in the intervention group

For a given value of the ICC in the control group, the intervention ICC can remain the same or it can change in a number of ways. In particular, the intervention ICC:

1. Remained the same as the control ICC,

2. Doubled, as a result of an appropriate increase in the between-cluster variance,

3. Doubled, as a result of an appropriate decrease in the within-cluster variance,

4. Halved, as a result of an appropriate decrease in the between-cluster variance,

5. Halved, as a result of an appropriate increase in the within-cluster variance.

Although it is only the between-cluster variance or the within-cluster variance that changes at any one time (not both), the changes involved are large ones. Hence these extreme scenarios should cover a range of findings.

### The number of clusters in each group

The number of clusters in each group was 6, 12 or 24. These figures reflect the small numbers of clusters recruited in many cluster RCTs and, coupled with the cluster sizes given below, they allowed alternative combinations of cluster size and number of clusters to be investigated for a given total trial size.

### The number of individuals in each cluster

The cluster size was 25, 50 or 100. Mean cluster sizes between 50 and 100 are not unusual in health services research trials, but there is enormous variation in cluster size, depending upon treatment area and type of cluster[1].

### Comparison of methods that allow for clustering

The first method utilises a standard procedure, where 'standard' has been taken to mean a 95% confidence interval quoted for continuous data in packages such as Stata [11], utilising a point estimate and a Huber-White (robust) cluster-adjusted standard error [12-14]. Bootstrapping was performed for the two other methods, as described in Davison and Hinkley [7]. Under both methods the sampling structure was maintained in a bootstrap replication by selecting *k *clusters with replacement from the treatment group and selecting *k *clusters with replacement from the control group – in other words, resampling of clusters was stratified by intervention group. Under method 1 all individuals within a resampled cluster were then selected. Under method 2 a second level of bootstrap was performed on individuals within clusters selected at level one. The difference between the two randomisation group means was then calculated for each method. This was repeated to give 1000 bootstrap estimates (estimates performed on the resampled data) of the treatment effect. A bias-corrected and accelerated (BC_a_) confidence interval was then estimated at the same nominal 95% level as for the robust method [8]. Given the nature of the BC_a _method, the resulting confidence interval need not be symmetric. The bootstrap methods are described in more detail below.

#### Method 1

Under this procedure (BS1), clusters are bootstrapped and each resampled cluster is kept intact. This method is utilised by Stata [11] when the cluster() option is added to the bootstrap command. Suppose that within a randomisation group, for each of k clusters, n responses are obtained, *y*_*ij*_, such that

*y*_*ij *_= *α*_*i *_+ *ε*_*ij *_    *i *= 1,...*k*;*j *= 1,...*n*.

The *α*_*i*_s are sampled randomly from the distribution *F*_*α *_and the *ε*_*ij*_s are independently sampled randomly from the distribution *F*_*ε*_.

E(*α*_*i*_) = 0;     (1)


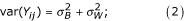



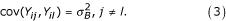


It can be shown that for the bootstrap estimates (with superscript asterisks):


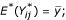



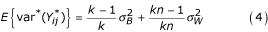



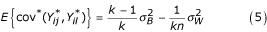


When compared with (2) and (3) it should be noted that the expected variance and covariance of the resampled outcome data are slightly biased downwards. However, an estimator such as the sample mean is strongly consistent (in that its bias is zero and its variance tends to zero as the total sample size approaches infinity); the level of bias is small unless the number of clusters becomes very small.

#### Method 2

An alternative method (BS2) involving resampling individuals as well as resampling whole clusters was also considered. This uses a first stage bootstrap applied to the estimated cluster means (sampling with replacement). The second stage, in which individuals are bootstrapped, involves resampling the deviations from the estimated cluster means. However, the estimated cluster means incorporate both within and between-cluster variability and any analysis that restricts itself to the cluster means will over-estimate the variance in these means [7]. By incorporating the deviations from the estimated cluster means we have, in effect, double-counted the within-cluster variance. Therefore, the cluster means were shrunk using Davison and Hinkley's shrinkage estimates, 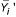 (see page 102 of their book):


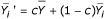


where c is given by


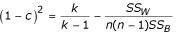
; if the right hand side is negative, it is reset to zero.

The variance of the adjusted cluster means, 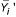, is then 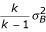.

The deviations from the estimated cluster means were also standardised to


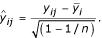


Finally, for all resampled clusters, the 'shrunken' mean is added to the standardised deviation for each resampled individual:


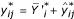


This method, with the rescaling procedures will in future be referred to as the BS2 method, or double bootstrap.

## Results

The primary focus of this work was on confidence interval coverage and rejection rates; the estimated confidence limits are, therefore, not presented but general inferences regarding the relative width of various confidence intervals can be made easily from the coverage and rejection rates presented below.

### Coverage rates for cost confidence intervals

As a summary, Tables 1 to 3 show coverage rates for each of the three methods of analysis for each sample size combination when averaged over the 15 parameter/distribution combinations for a given control group ICC – that is, three distributional assumptions for each of five ICC combinations.

Issues specific to variance or distribution combinations are presented below. In the three tables, within each box the first number represents the coverage of the robust confidence interval, the second represents the coverage of the BS1 method whilst the third figure represents that of the BS2 method. A number of results are immediately apparent:

• All three methods produce coverage of less than 95%, the nominal level,

• All three methods appear to be consistent with respect to the impact of the number of clusters per arm. In other words, as the number of clusters per arm increases, the observed coverage approaches 95% for each method,

• The coverage of the robust method is within approximately 1.5% of 95%,

• The BS1 method is always outperformed by the other two methods. In other words the other two methods always achieve coverage that is closer to 95%,

• The BS2 method performs much better than the BS1 method but never as well as the robust method,

• As the ICC in the control group increases (that is, across tables) the robust method performs slightly worse whilst the performances of the bootstrap methods are noticeably poorer,

• When examining numbers along the diagonal in each figure (that is, for equivalent total sample sizes), the bootstrap methods perform much better for a large number of clusters and small cluster size compared with vice-versa. This is probably due to the slight downward bias in the second moments; the degree of bias is an inverse function of the number of clusters.

From the results in these tables there does not appear to be much to commend the bootstrap, since both bootstrap methods are always outperformed by the robust method. Moreover, when examining the confidence interval coverage results split by distribution and variance combination (results not shown), there was only one parameter combination for which the robust method was outperformed and this was by an amount that was consistent with Monte Carlo sampling error. However, the rejection rates for confidence intervals were split to ascertain if any method was noticeably better at taking account of the skewness in the data.

### Rejection rates for confidence intervals

When examining the rejection rates for six clusters of size 25 (results not shown), those of both bootstrap methods virtually always exceeded those under the robust method. The exceptions were for a control ICC of 0.01 when the within-cluster variance increased as a result of the intervention for two of the distributional combinations. For the third distributional combination the rejection rates were the same. Since the individual level data were always lognormally distributed (reflecting typical cost data at this level), an increase in the within-cluster variance for lognormally distributed data is likely to have a large effect on skewness, provided the ICC is not too large (which would dilute the effect of within-cluster factors).

Thus, if the desired criterion is ability to match the nominal 2.5% rejection rate in any given direction, there are occasions when one or both bootstrap methods outperform the robust method in terms of the proportion of lower rejections. In particular, this appeared to happen when the ICC was moderate to large, together with an extremely skewed distribution of the treatment effect, typically achieved by a large change in the ICC and something other than a normal, normal (N, N) distribution combination. Under these circumstances the distribution of the treatment effect is most skewed, since the cluster means in the intervention group are exhibiting large skewness by way of a lognormal distribution whose (already moderate to large) variance has doubled. However, this must be balanced against the poor performance of the bootstrap methods in terms of the proportion of upper rejections, which tended to be particularly high compared with those from the robust method.

### Larger cluster size

With a cluster size of 50 or 100 (results not shown), similar results were seen to those above, in that a large difference in the absolute value of the ICC together with extremely skewed distributions were required for the double bootstrap to achieve a rejection rate closer to the nominal level than the robust method. In particular, increases in the between-cluster variance accompanied by skewed distributions at the between-cluster level typically caused the double bootstrap to give better lower rejection rates.

### Larger number of clusters

For 12 clusters of size 25 per arm (results not shown), all three methods performed better than for an equivalent sample size with fewer clusters per arm (6 clusters of size 50). There were very few instances in which the rejection rate exceeded that for 6 clusters of size 50. Even the BS1 method appeared to perform more consistently than when there were only six clusters per arm. Although it was still always outperformed by the BS2 and robust methods, its maximum rejection rate was 10.26%, compared with 14.24%. As before, when the ICC was very small (that is, most of the variability was within clusters), the double bootstrap method typically only outperformed the robust method when the within-cluster variance increased. In addition, for larger values of the ICC, changes in the between-cluster variance or the within-cluster variance could result in the double bootstrap outperforming the robust method, confirming the results obtained for six clusters of size 100. Lastly, normal distributions at the between-cluster level in both arms were sufficient to ensure that the robust method always performed better than the bootstrap.

### Larger number of clusters and larger cluster size

All the trends identified in previous sections were replicated for 24 clusters of size 25 (see Tables 4, 5, 6). Interestingly, for ICC = 0.25, this sample size combination produced the largest number of occasions on which the BS2 method outperformed the robust method, namely eight. However, this should be balanced against the continued better coverage of the robust method overall.

## Discussion

This work constitutes early stages in the further research that has been advocated to identify appropriate approaches to the analysis of cost outcomes from cluster RCTs [10]. The ICCs used in the present simulations were comparable to those estimated for costs in this previous study, but highly variable ICCs for costs at different levels and the magnitude of patient costs relative to total costs have both been emphasised as important issues [10]. Thus, decisions as to whether any of the scenarios investigated here are relevant to future trials will depend, in part, upon the issue of which cost component is most important.

### Limitations

The main limitation of the work presented here is the lack of empirical data to inform the modelling. Empirical data on costs from cluster RCTs are required to investigate whether the bootstrap method should be evaluated in the presence of even more highly skewed data. The bootstrap methods of Davison and Hinkley also require testing in trials with non-constant cluster size. For the single bootstrap, in the presence of a non-constant cluster size, each bootstrap sample of clusters will have a different composition. The sample mean will exhibit a different degree of variability depending upon, for example, whether the bootstrap sample has happened to select many large or many small clusters. The result may be incorrect inferences about the variability in the sample mean. Moderate variability in cluster size or a large number of clusters might not be expected to have a large effect upon the estimated confidence limits, but again the researcher would have to be cautious. Whilst the double bootstrap can address this issue, this version resamples only the deviations from the cluster mean of an individual's 'own' cluster, potentially omitting valuable statistical information [7]. However, more complex bootstrap methods such as those of Rao and Wu and Carpenter *et al *do not suffer from this restriction and future work should allow the cluster size to vary [9,15,16].

### Future research

Despite these limitations, the results from the simulations present a coherent picture of the relative strengths of the methods of analysis that were compared. They also show that methods of analysis that can deal adequately with trial data incorporating a small number of clusters must be developed and investigated. For cost data the robust method gave confidence intervals with broadly correct coverage when the cluster size was constant. The Huber-White estimator can take account of non-constant cluster size, so future work should address its ability to give acceptable confidence intervals when data are skewed and the cluster size varies within the trial.

## Conclusions

There are a number of general points that can be drawn from these results. First, when the between-cluster distribution is normal in both treatment arms, there is virtually no evidence in favour of using a bootstrap method. Second, when the ICC takes values of about 0.1 or greater, the double bootstrap can give a lower rejection rate which is closer to the nominal level than that achieved by the robust method, particularly when the between-cluster distributions are skewed. However, this is only common when the ICC changes as a result of the intervention. Third, the downward bias in the second moments of the bootstrap methods is particularly problematic. In general, 24 clusters per treatment arm is probably the minimum number for which one would even begin to consider the bootstrap in preference to traditional robust methods, for the parameter combinations investigated here. At least this number of cluster and extremely skewed data would be necessary for the bootstrap to approximate the results from the robust method with any consistency. The likelihood of such a scenario will clearly vary, but in any case these simulations have brought out a very important issue: a normal distribution for the cluster means is usually sufficient to eliminate the bootstrap from any consideration, regardless of the skewness of the individual data.

This work has related to skewed cost (or potentially clinical) data. Whilst this is of interest, policymakers are more often interested in cost-effectiveness data. These have usually involved ratio statistics, which often cause major problems for traditional estimators. Future work should, therefore, investigate how well the cluster bootstrap deals with cost-effectiveness data and hence address the question of whether the disadvantages of the cluster bootstrap identified here are outweighed by its potential advantages in dealing with ratio statistics.

## Competing interests

The author(s) declare that they have no competing interests.

## Authors' contributions

TNF conceived of the study, wrote and conducted the simulation programs and drafted the manuscript.

TJP participated in the design and coordination of the study, read, critically reviewed and contributed to drafts of the manuscript.

## Pre-publication history

The pre-publication history for this paper can be accessed here:



## Figures and Tables

**Table 1:** Observed coverage (%) for robust, BS1 and BS2 methods of analysis (Control ICC = 0.01)

		**Cluster Size**		
		**25**	**50**	**100**
**Number of clusters per arm**	**6**	94.1	93.8	94.0
		88.2	88.2	88.3
		93.2	93.1	92.7
	
	**12**	94.6	94.5	
		91.1	91.3	
		93.4	93.3	
	
	**24**	94.8		
		92.7		
		93.7		

**Table 2:** Observed coverage (%) for robust, BS1 and BS2 methods of analysis (Control ICC = 0.1)

		**Cluster Size**		
		**25**	**50**	**100**
**Number of clusters per arm**	**6**	93.9	93.9	93.8
		87.6	87.3	86.9
		90.9	90.6	90.3
	
	**12**	94.5	94.4	
		90.5	90.2	
		91.8	91.6	
	
	**24**	94.8		
		92.1		
		92.6		

**Table 3:** Observed coverage (%) for robust, BS1 and BS2 methods of analysis (Control ICC = 0.25)

		**Cluster Size**		
		**25**	**50**	**100**
**Number of clusters per arm**	**6**	93.6	93.5	93.5
		86.9	86.6	86.4
		90.1	89.9	89.6
	
	**12**	94.3	94.2	
		89.9	89.6	
		91.3	91.1	
	
	**24**	94.6		
		91.5		
		92.1		

**Table 4:** Control ICC = 0.01, 24 clusters of size 25 per arm.

**How does the ICC change as a result of the intervention?**		**Between-cluster distribution combination**^1^	**Rejection Rate (%)**^2^					
			
			**Huber-White**		**Single Bootstrap**		**Double Bootstrap**	
			
			**Lower**	**Upper**	**Lower**	**Upper**	**Lower**	**Upper**
**No change**	**-**	N, N	2.65	2.45	3.55	3.46	3.11	3.08
		LN, LN	2.57	2.64	3.80	3.68	3.25	3.27
		N, LN	2.42	2.76	3.64	3.67	3.21	3.39

**Double**	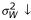	N, N	3.08	2.35	3.65	3.57	3.13	3.37
		LN, LN	2.88	2.37	4.00	3.75	3.36	3.40
		N, LN	2.84	2.46	3.93	3.45	3.29	3.27
	
	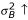	N, N	2.55	2.59	3.38	3.40	3.26	2.99
		LN, LN	2.44	2.82	3.91	4.05	3.53	3.55
		N, LN	2.07	3.00	3.50	3.77	3.04	3.41

**Halve**	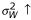	N, N	**1.97**	3.27	3.23	3.97	**2.79**	3.30
		LN, LN	2.10	3.03	3.49	3.83	2.93	3.04
	
		N, LN	**1.92**	**3.20**	3.24	3.77	**2.87**	**2.95**
	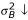	N, N	2.66	2.59	3.66	3.58	3.06	3.06
		LN, LN	2.54	2.46	3.63	3.71	3.00	3.14
		N, LN	2.38	2.57	3.43	3.51	2.87	3.05

^1 ^Control arm is first, followed by intervention arm. N, N denotes normal distribution in both arms; LN, LN denotes lognormal distribution in both arms; N, LN denotes normal distribution in control arm and lognormal distribution in intervention arm

^2 ^Entries in bold indicate where double bootstrap method achieved rejection rate closer to nominal 2.5% than Huber-White method.

**Table 5:** Control ICC = 0.1, 24 clusters of size 25 per arm.

**How does the ICC change as a result of the intervention?**		**Between-cluster distribution combination**^1^	**Rejection Rate (%)**^2^					
			
			**Huber-White**		**Single Bootstrap**		**Double Bootstrap**	
			
			**Lower**	**Upper**	**Lower**	**Upper**	**Lower**	**Upper**
**No change**	**-**	N, N	2.70	2.72	3.25	3.34	3.00	3.16
		LN, LN	2.47	2.38	4.95	4.92	4.53	4.47
		N, LN	**1.48**	3.64	3.23	4.04	**2.85**	4.05

**Double**	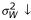	N, N	2.66	2.53	3.12	3.19	2.85	3.13
		LN, LN	2.28	2.36	4.99	5.07	4.60	4.63
	
		N, LN	**1.59**	3.70	3.49	4.25	**3.04**	4.25
	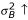	N, N	2.43	2.77	2.94	3.38	2.71	3.05
		LN, LN	1.42	3.90	4.45	5.94	3.99	5.49
		N, LN	**1.10**	**4.97**	3.38	5.00	**2.91**	**4.95**

**Halve**	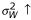	N, N	2.38	2.72	3.17	3.30	3.18	3.03
		LN, LN	2.25	2.50	4.48	4.61	4.15	4.14
		N, LN	**1.65**	3.77	3.47	4.02	**3.10**	3.89
	
	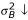	N, N	2.73	2.48	3.33	3.16	3.10	3.09
		LN, LN	3.34	1.69	5.26	4.06	4.91	3.64
		N, LN	2.20	2.95	3.61	3.56	3.17	3.49

^1 ^Control arm is first, followed by intervention arm. N, N denotes normal distribution in both arms; LN, LN denotes lognormal distribution in both arms; N, LN denotes normal distribution in control arm and lognormal distribution in intervention arm

^2 ^Entries in bold indicate where double bootstrap method achieved rejection rate closer to nominal 2.5% than Huber-White method.

**Table 6:** Control ICC = 0.25, 24 clusters of size 25 per arm.

**How does the ICC change as a result of the intervention?**		**Between-cluster distribution combination**^1^	**Rejection Rate (%)**^2^					
			
			**Huber-White**		**Single Bootstrap**		**Double Bootstrap**	
			
			**Lower**	**Upper**	**Lower**	**Upper**	**Lower**	**Upper**
**No change**	**-**	N, N	2.76	2.73	3.31	3.27	3.12	3.06
		LN, LN	2.28	2.32	5.77	5.55	5.30	5.16
		N, LN	**1.38**	4.08	3.42	4.43	**3.00**	4.30

**Double**	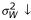	N, N	2.84	2.66	3.30	3.20	2.91	3.09
		LN, LN	2.03	2.17	5.55	5.81	5.07	5.25
		N, LN	**1.41**	4.13	3.60	4.45	**3.18**	4.21
	
	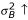	N, N	2.63	2.74	3.12	3.22	2.88	2.98
		LN, LN	**0.84**	5.38	3.84	7.90	**3.43**	7.39
		N, LN	**0.71**	**6.53**	3.25	6.20	**2.94**	**5.89**

**Halve**	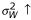	N, N	2.56	2.65	3.16	3.19	2.99	2.84
		LN, LN	1.98	2.38	4.96	5.32	4.57	4.70
		N, LN	**1.47**	**4.21**	3.45	4.41	**3.12**	**4.11**
	
	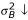	N, N	2.56	2.79	3.09	3.25	2.91	3.10
		LN, LN	4.26	1.39	6.70	4.54	6.16	4.15
		N, LN	**1.79**	3.36	3.24	3.73	**2.95**	3.57

^1 ^Control arm is first, followed by intervention arm. N, N denotes normal distribution in both arms; LN, LN denotes lognormal distribution in both arms; N, LN denotes normal distribution in control arm and lognormal distribution in intervention arm

^2 ^Entries in bold indicate where double bootstrap method achieved rejection rate closer to nominal 2.5% than Huber-White method.
